# The IgA nephropathy Biobank. An important starting point for the genetic dissection of a complex trait

**DOI:** 10.1186/1471-2369-6-14

**Published:** 2005-12-05

**Authors:** Francesco P Schena, Giuseppina Cerullo, Diletta D Torres, Francesco Scolari, Marina Foramitti, Antonio Amoroso, Doroti Pirulli, Jürgen Floege, Peter R Mertens, Klaus Zerres, Efstathios Alexopoulos, Dimitrios Kirmizis, Leopoldo Zelante, Luigi Bisceglia, Gian M Ghiggeri, Giovanni M Frascà

**Affiliations:** 1 Renal Unit, University of Bari, Italy; 2 Renal Unit, University of Brescia, Italy; 3 Dipartimento di Genetica, Biologia e Biochimica, University of Torino, Italy; 4 Dipartimento di Biochimica, Biofisica e Chimica delle Macromolecole, University of Trieste, Italy; 5 Division of Nephrology and Immunology, University of Aachen, Germany; 6 Institute of Human Genetics, University of Aachen, Germany; 7 Renal Unit, Aristotelian University of Thessaloniki, Greece; 8 Genetic Unit, IRCCS-Casa Sollievo della Sofferenza, San Giovanni Rotondo, Foggia, Italy; 9 Renal Unit, Ospedale Gaslini, Genova, Italy; 10 Renal Unit, University of Bologna, Italy

## Abstract

**Background:**

IgA nephropathy (IgAN) or Berger's disease, is the most common glomerulonephritis in the world diagnosed in renal biopsied patients. The involvement of genetic factors in the pathogenesis of the IgAN is evidenced by ethnic and geographic variations in prevalence, familial clustering in isolated populations, familial aggregation and by the identification of a genetic linkage to locus IGAN1 mapped on 6q22–23. This study seems to imply a single major locus, but the hypothesis of multiple interacting loci or genetic heterogeneity cannot be ruled out. The organization of a multi-centre Biobank for the collection of biological samples and clinical data from IgAN patients and relatives is an important starting point for the identification of the disease susceptibility genes.

**Description:**

The IgAN Consortium organized a Biobank, recruiting IgAN patients and relatives following a common protocol. A website was constructed to allow scientific information to be shared between partners and to divulge obtained data (URL: ). The electronic database, the core of the website includes data concerning the subjects enrolled. A search page gives open access to the database and allows groups of patients to be selected according to their clinical characteristics. DNA samples of IgAN patients and relatives belonging to 72 multiplex extended pedigrees were collected. Moreover, 159 trios (sons/daughters affected and healthy parents), 1068 patients with biopsy-proven IgAN and 1040 healthy subjects were included in the IgAN Consortium Biobank. Some valuable and statistically productive genetic studies have been launched within the 5^th ^Framework Programme 1998–2002 of the European project No. QLG1-2000-00464 and preliminary data have been published in "Technology Marketplace" website: .

**Conclusion:**

The first world IgAN Biobank with a readily accessible database has been constituted. The knowledge gained from the study of Mendelian diseases has shown that the genetic dissection of a complex trait is more powerful when combined linkage-based, association-based, and sequence-based approaches are performed. This Biobank continuously expanded contains a sample size of adequately matched IgAN patients and healthy subjects, extended multiplex pedigrees, parent-child trios, thus permitting the combined genetic approaches with collaborative studies.

## Background

IgA nephropathy (IgAN) or Berger's disease is the most common glomerulonephritis worldwide in patients undergoing renal biopsy. Diagnosis is based on the occurrence of mesangial deposits of IgA in the glomeruli in the presence of recurrent episodes of intra-infectious macroscopic hematuria or persistent microscopic hematuria and/or proteinuria. The frequency of this disease is renal biopsy policy-dependent [1]. In some countries, the majority of patients with recurrent macroscopic hematuria undergo renal biopsy only in the presence of proteinuria or mild renal insufficiency. In Eastern countries, however, many young subjects with persistent microscopic hematuria and mild proteinuria often receive renal biopsy. This is not the policy in most Western countries, in addition often asymptomatic subjects with persistent urinary abnormalities (microhematuria and/or proteinuria) decline renal biopsy. This large variability in symptoms induces different approaches in performing renal biopsy. Nevertheless, renal biopsy practice is not the sole cause of the different disease worldwide prevalence. Striking ethnic variation in prevalence [2-6] along with familial clustering [7-9] are suggestive of an important role of the genetic component in the pathogenesis of the disease. IgAN may occur in a sporadic or a familial form according to the clinical evidence that one or more subjects belonging to the same family are affected by biopsy-proven IgAN [10-12]. Moreover, sub-clinical renal abnormalities are often evidenced among relatives of IgAN patients [9,11,13].

Genetic analysis of large pedigrees of IgAN families is considered the most promising approach to identify IgAN susceptibility genes. The genome wide scanning in 30 large extended IgAN multiplex families (24 from Italy and 6 from United States) was performed by Gharavi et al [14]. This study identified the locus, called IGAN1, located on chromosome 6q22–23 in linkage with IgAN. It yielded a significant peak Lod-score of 5.6, with 60% of families linked, assuming an autosomal dominant mode of inheritance with reduced penetrance. This mode of inheritance of familial IgAN is more consistent with the involvement of a single gene with a large effect located in IGAN1. Nevertheless, multifactorial determination, with the interplay of many genes, each conferring a small effect cannot be excluded. The knowledge gained from the study of Mendelian diseases has shown that genetic dissection of a complex trait is more powerful when combined linkage-based, association-based, and sequence-based approaches are performed [15]. Association-based studies are possible when a large sample size of adequately matched IgAN patients and healthy subjects is collected. Prior insight into the pathogenesis of the disease allows to establish the candidate genes to be studied according to their known or suspected function. Over the past decade, a shift has occurred away from case-control association studies, towards family-based designs in which extended pedigrees, relative-pairs and parents-child trios are used to test for association. For this purpose, different powerful and sensitive methods of analysis have been developed, most of which are based on the trasmission/disequilibrium test (TDT) [16].

Considering the possibility of identifying IgAN susceptibility genes a group of European scientists, specialists in this disease, constituted an IgAN Consortium and organized a Biobank project recruiting biological samples and clinical data from IgAN patients and relatives. This report describes the creation of the first Biobank with a readily accessible database of the most common worldwide glomerulonephritis, a rare disease which requires a multi-centre project organization.

## Construction and content

The IgAN Consortium was based on the constitution of a collaborative study group including expert nephrologists from Italy (FPS, FS, GMF), Germany (JF) and Greece (EA), geneticists from Italy (AA, LB, GC, GMG) and Germany (KZ). The European project No. QLG1-2000-00464 was funded by the 5^th ^Framework Programme 1998–2002. Additional funds were obtained in Italy from MIUR (Ministero dell'Istruzione, Universita' e Ricerca PRIN 2001-067748; L.488/92 Cluster 03; FIRB 2001-RBNE013JYN).

**Figure 1:**
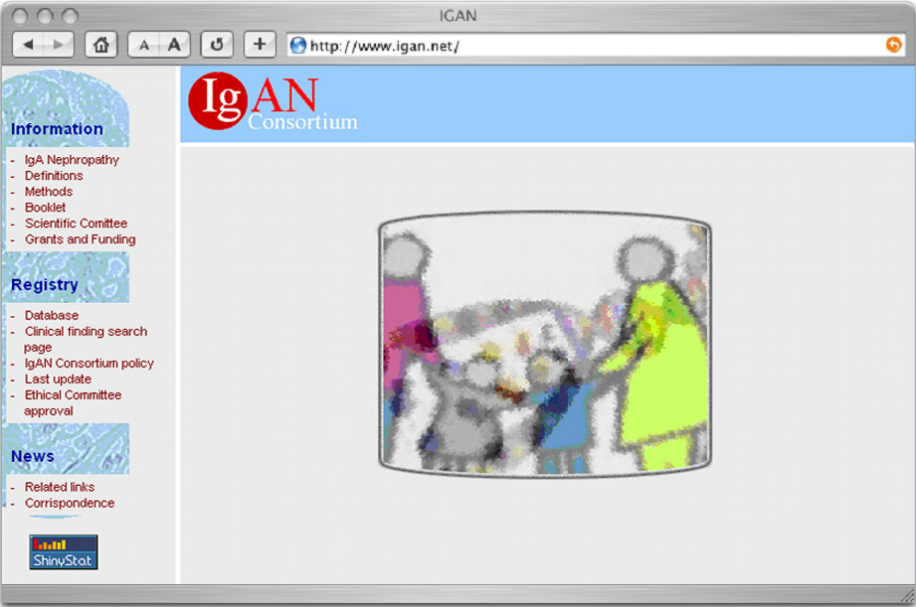
IgAN Consortium website structure.

The main aim of the project was to constitute a genomic DNA bank of well characterized IgAN patients and their relatives from three different European countries (Germany, Greece and Italy) to be used for genetic studies. The following objectives were reached:

1. a) Definition of a common protocol for the diagnosis of familial, suspected and sporadic IgAN. IgAN was diagnosed on the basis of either recurrent episodes of macroscopic hematuria concomitant with upper respiratory tract and other infections or permanent microscopic hematuria with/out proteinuria. Mesangial glomerular IgA deposits in the renal biopsy and absence of systemic or hepatic diseases confirmed the diagnosis. Relatives of at least three generations received urinalysis. All relatives with persistent microscopic hematuria received an Addis count under contrast phase microscopy (glomerular hematuria was confirmed at least three times) and renal ultrasound. Relatives with suspected IgAN were informed and received renal biopsy to confirm the diagnosis. All subjects were categorized as: (I) affected subjects with biopsy-proven disease; (II) subjects probably affected by IgAN when persistent microscopic hematuria without biopsy-proven glomerulonephritis, or chronic renal insufficiency or ESRD treated with hemo or peritoneal dialysis, or renal transplantation after biopsy-proven IgAN or unknown glomerulonephritis, or death caused by chronic uremia, occurred; (III) non-affected subjects in the absence of urinary abnormalities; (IV) subjects with unknown status, who refused participation or in the absence of clinical information and laboratory findings. Familial IgAN was diagnosed when at least 2 family members had biopsy-proven IgAN. Suspected IgAN families were considered those in which one subject was a biopsy-proven IgAN patient and others were probably affected, were awaiting or refused renal biopsy, or had urinary abnormalities that did not justify its execution. They were checked systematically once a year. Sporadic IgAN was diagnosed when the disease occurred only in the patient and relatives were negative by urinalysis. b) Layout of a booklet containing information, letter of informed consent and instructions for the collection of blood samples. c) Definition of a data sheet for the collection of personal data, clinical and laboratory findings.

2. Constitution of the Biobank. Blood samples from all the participants after informed consent (disclosing information about the research objectives, benefits and risks) were obtained. Thirty ml of peripheral blood was drawn from each subject aged more than 15 years and 15 ml from each younger subject. Plasma and serum samples were stored at -80°C in order to be used for the biochemical (inflammatory cytokines and chemokines) and immunological parameter measurements (complement components, IgA serum level, IgA galactosylation) as required. EDTA tubes were used to collect blood samples for DNA isolation. The EDTA anticoagulated blood sample from each subject was divided into three aliquots, two of which were processed for DNA extraction, in a separate manner, using commercial kits. The first DNA sample was stored in ethanol 75% at 4°C, in the form of a pellet. The second one was suspended in deionised nuclease free water and subdivided into two aliquots ready to use. They were independently stored at 4°C and at -20°C. Storage at 4°C prevents degradation due to the mechanical stress of freezing and thawing and storage at -20°C inhibits possible DNase activity. This method was adopted to guarantee an adequate quality DNA sample and long term storage. Moreover, the last aliquot of whole blood was stored at -20°C in order to isolate a DNA sample whenever needed. DNA was isolated in the Bari, Trieste, Genova and Aachen laboratories. F.P.S., F.S., A.A., J.F., E.A. oversaw the accuracy of the DNA sample collection. All the IgAN patients and relatives, from whom DNA samples were collected, were listed in the database and were available to the IgAN Consortium partners and to the investigators proposing collaborative studies. The protocol was approved by the local Ethical Committees. The documents regarding each local Ethical Committee who have given approval for the study, in their original language, have been disclosed as a window in the IgAN Consortium website [17], purposely constructed for this study.

3. Construction of a website to share scientific information between partners and to divulge obtained data to the community. The IgAN Consortium website was designed and realized by Apulia Biotech (Valenzano, Bari, Italy) and is managed by Altanet SRL (Altamura, Bari, Italy) internet provider. The website includes three sections: (a) Information, (b) Registry and (c) News (fig. 1) all readily accessible for public vision. Section (a) contains guidelines for the collection of blood samples, information about the disease and the informed consent form to be compiled. Section (b) encloses the electronic database for the collection of all personal and clinical data of each enrolled subject. The IgAN Consortium partners are given personal passwords to insert and modify information included in the database. This section is also provided with a search page (clinical finding search page) that gives free access to the database. Groups of IgAN patients can be selected according to their specific characteristics. The clinical history can be viewed observing the confidentiality of each subject. Important information about IgAN Consortium policy for collaborative studies are given. This section is periodically updated in all its parts. Section (c) is devoted to public dissemination of achieved data. It presently includes the report of three years of activities.

4. Construction of an electronic database, for the collection of personal data, clinical, histological and laboratory findings. The database was developed in Filemaker Pro 5.0 obtaining a powerful cross platform relational database. The website interface was realized by using Lasso by Omnipilot Software Inc as the local data markup language (LDML) that connected our HyperText Markup Language (HTML) pages to the Filemaker database. 4D Webstar was used as Internet server. It encloses the list of IgAN patients and relatives enrolled, and the familial IgAN pedigrees. Pedigrees were drawn using the software Cyrillic 2.1. Each collected family and subject was univocally identified by a sequence of three numeric codes: partner unit, family and subject code. Each partner identified a staff member, responsible for the attribution of the codes. Progressive numeric codes for families and subjects were assigned according to the time of enrolment in this study. All the data collected for each enrolled subject at the onset of the disease, at the time of the renal biopsy and at the available follow up are included in the database. Serum creatinine (sCR) and daily proteinuria were obtained from IgAN patients at different times. Daily proteinuria was defined as mild (<1 g), moderate (1–3 g) and severe (>3 g). Renal function was evaluated by creatinine clearance obtained using the Cockcroft formula [18]. According to the K-DOQI guidelines [19] the stages of chronic kidney disease were classified as kidney damage with normal renal function (creatinine clearance ≥ 90 ml/min), mild (60–89 ml/min), moderate (30–59 ml/min) and severe impaired renal function (15–29 ml/min) and kidney failure (<15 ml/min or dialysis). According to the 2003 ESH/ESC hypertension guidelines [20], subjects were defined hypertensives when blood pressure was above 130/85 mmHg or when they received anti-hypertensive drugs. Three outcome measures were considered in the follow up of the IgAN patients: chronic renal insufficiency, dialysis and renal transplant. Renal biopsy specimens were scored considering the severity of glomerular, tubulointerstitial and vascular lesions. According to the WHO classification 3 histological grades (G) were identified: a) G1 (mild disease); b) G2 (moderate disease); c) G3 (severe disease) [21,22]. In the search page of the website Registry-section the biopsy-proven IgAN patients (BP-IgAN) may be selected according to these clinical and laboratory characteristics at the time of the renal biopsy or according to the main outcome measures. The result page included three important links: the "*pedigree" *field allows the visualization of the familial IgAN cases; the "*info" *field introduces the complete clinical history of each patient and the "*follow up" *field links directly to the data at different follow up times for each patient.

A medical geneticist collaborating with each nephrology unit involved in the project offered genetic counselling to IgAN patients and relatives. The main purpose of the counselling is to give information on a complex genetic disease such as IgAN and to illustrate the Biobank objectives, underlining that the genetic tests will not give an immediate answer to the possibility of developing the disease or not. The clinicians explained to all patients the potential familial incidence of the disease and invited their relatives to the genetic counselling session in which a clinical screening was proposed for all relatives. In addition, they were informed on the possibility to be enrolled in this Biobank. For this purpose, the aims of the project were discussed in lay terms with respect to the risk for the involved subjects. It was clearly stated that the subjects tested would not benefit personally from the genetic studies. Financial rewards were not offered to encourage participation. Patients and relatives were informed that the DNA would be used only for the present project i.e. genetic dissection of IgAN. Moreover, a detailed description of the genetic studies to be carried out was given. Informed consent was signed after the genetic counselling.

Normal subjects were recruited among healthy blood donors with negative urinalysis and no history of renal disease, diabetes, hypertension or metabolic disorders. They were matched for age, gender and ethnicity to the IgAN patient population enrolled. They were scrupulously informed about the aim of the study. They gave a written informed consent for DNA collection and for the handling of their genetic and personal data for the present project. The collection of DNA samples is still underway. The IgAN Consortium collected DNA samples from IgAN patients and relatives belonging to at least three generations, with particular attention paid to the collection of all first degree relatives. DNA samples, collected from the research units are listed in table 1. All the subjects enrolled were of Caucasian origin and belonging to different European geographic area: North and South Italy (Lombardia, Piemonte, Friuli Venezia Giulia and Puglia region), Greece (Thessaloniki), Germany (Nordrhein, Westfalia region). DNA samples from 72 multiplex IgAN families, 63 suspected IgAN families, 1068 patients with biopsy-proven IgAN were obtained. The number of collected relatives for each family enrolled was different. Fifty-one IgAN families have less than 10 relatives, 17 include a number of relatives ranging from 10 to 20 subjects and finally 4 IgAN families have more than 20 relatives. Forty-nine suspected IgAN families have less than 10, 13 less than 20 and 1 family has more than 20 relatives. A large sample size was obtained in the form of trios (son/daughter affected and healthy parents). A large number of healthy blood donors adequately matched for age, gender and origin to the patient populations were recruited as controls in each area.

Seventy-two IgAN families included DNA samples from 94 pairs of biopsy proven IgAN patients and a total number of 2573 pairs of relatives. Table 2 shows the distribution of pairs of relatives collected from the 72 IgAN families according to genetic relationship and disease.

**Figure 2:**
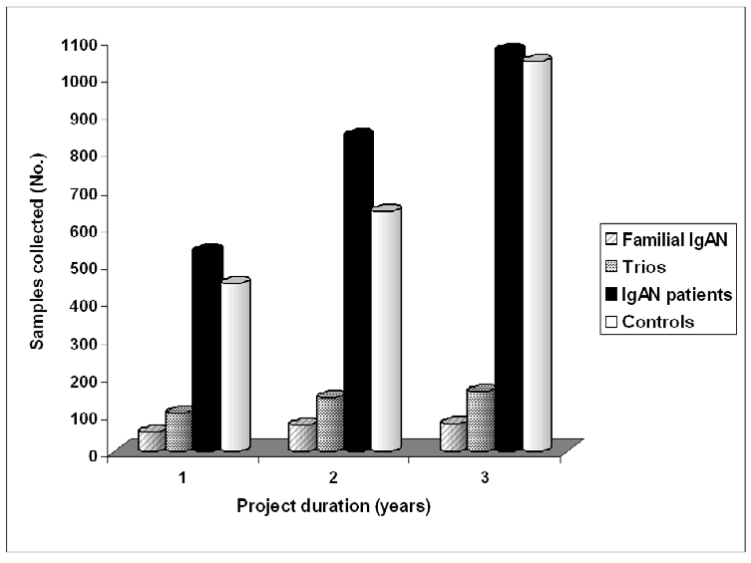
DNA samples collected from the beginning of the IgAN Consortium European project.

A gradually increasing number of DNA samples collected during the first three years of our project (from beginning of October 2000 to the end of December 2003) is shown in figure 2. Some valuable and statistically productive genetic studies have been launched within the 5^th ^Framework Programme 1998–2002 of the European project No. QLG1-2000-00464. Case-control and family-based association studies investigating the possible role of some Th1/Th2/Th3/T_R_-type, and of monocyte/macrophage cytokines gene polymorphisms and Core1 β 1,3- Galactosyltransferase (β1.3 C1GALT1) gene are presently under way. Some preliminary data are publicly available in the "Technology Marketplace" website [23]. Further investigations needs to strengthen the data obtained.

## Utility and discussion

We have described the constitution of the European IgAN Biobank which is the first DNA bank created in the world by the multi-centre collaborative project for this disease. The main aim of this project was to organize a genomic DNA bank of biopsy-proven IgAN patients and their family members belonging to three different geographic areas of Europe (Germany, Greece and Italy). The Biobank has taken care of ethical aspects since the collection and storage of personal and genetic information follow the indications of the Council of Europe, as stated in the Convention on human rights and biomedicine (Nov 19, 1996), and those of UNESCO, as reported in the Declaration on human genome (Nov 11, 1997). In addition, the handling of personal and genetic data collected follow the indications and norms existing in each participating country.

The IgAN Consortium has set up a website to allow the partners to share the scientific information and for public dissemination of all the information related to the disease and new achieved data [17]. The accessible database without restriction permits to share clinical data of IgAN patients with other participating centres. A search page supports the investigators in selecting IgAN patient groups with respect to specific clinical data to perform prospective and retrospective studies.

Studies that seek to identify disease genes can be divided into two categories: linkage and association studies. Linkage studies speculate on the co-segregation of marker alleles and disease of interest in multiplex families while association studies detect linkage disequilibrium between marker and disease loci using case-control and/or family-based designs. In the simplest design, genetic variant frequencies of exposure are compared to diseased cases and non diseased controls. This approach, highly effective in detecting genes of modest effect, is susceptible to "population stratification bias" arising from differences in the genetic background of cases and controls. The distribution of alleles in a population is related to the ethnic and social background and geographical origin of their parents. There is a great debate on the possibility of analysing isolated populations for genetic studies, which may be homogeneous such as Finnish, Icelanders, Sardinians, or heterogeneous populations such as in the case of the UK Biobank [24-28]. Researchers reason that in a population such as Iceland's (270,000 individuals), which expanded from a relatively small number of founders and that did not experience significant immigration, there should be fewer opportunities for particular markers and disease genes to have become separated down the generations. On the other hand some investigators recently provide results that isolated populations are unlikely to be very different from more mixed populations in terms of linkage disequilibrium [29]. Tom Meade of the Medical Research Council (MRC) in Britain, acknowledged that heterogeneous populations will have some advantage because the results will be representative of the population as a whole [25]. The family-based approach in which controls are represented by healthy family members has attracted considerable attention since it minimizes population stratification bias and it avoids making erroneous conclusion. In our Biobank we proposed to collect DNA samples from patients, their relatives and an adequate number of controls of different geographic areas to enable each research unit, independently, to perform case-control and/or family-based association studies in a population belonging to a restricted area. Whenever statistical evidence for a genetic association was obtained, the same data would be checked for the populations collected in other geographic areas. This organization could be highly representative of the IgAN genetic background and more powerful in the detection of weak genetic effects.

It is the policy of the IgAN Consortium to favour further collaborative genetic studies. Other investigators not belonging to the IgAN Consortium, who would like to have access to the DNA Bank for genetic studies, must submit the study design to the scientific committee represented by the principal investigators of the IgAN Consortium research units (F.P.S., F.S., A.A., J.F., E.A., K.Z., L.Z.) [see Additional file 1] and must agree to the terms for data publication. Adequate procedures are used to minimize inadvertent release of personal information and to guarantee confidentiality of genetic results, such as disease genetic predisposition or family relationships (non-paternity, adoption). All results published must not reveal the patient's identity.

The availability of genetic material from participants in large epidemiological studies represents an invaluable resource for exploring genetic and environment influence on disease risk. In fact, the world's largest study on cancer is the multi-centre European Prospective Investigation into Cancer and Nutrition (EPIC), which started recruiting in the early 1990's and has now available more than 500,000 individuals [30]. This has allowed the project to move onto the study of genetics since its sample includes 350 cases of colon cancer and nearly 2000 of breast cancer. Our data collection of more than 1000 individuals with IgAN is close to the above mentioned EPIC data for each disease. Robert Hoover, director of epidemiology and biostatistics at the National Cancer Institution at Bethesda, Maryland, defines a large epidemiological study as a study which generates 1000 to 1500 individuals with a particular cancer in a reasonable time [26]. Keeping this in mind we collected DNA samples from more than 1000 IgAN patients. However, our Biobank will continue to enrol additional patients in the future for potential stratification of our population with respect to specific phenotypes thus promoting correlations with discovered genotypes.

During these last ten years many biobanks have been organized but there is no system for comprehensive registration of the DNA banks. Surely many case-control and family-based association studies will originate from these DNA banks in the future. The IgAN Consortium Biobank has allowed us to carry out a linkage study and various case-control and family-based association studies whose investigation is currently under way. We think that the inclusion of all DNA banks in a public registry might create many advantages for scientists and patients as follows: I) people involved in the scientific and clinical activity of a certain diseases may have a point of reference; II) network of scientists involved in basic and clinical research may obtain many advantages by the public registry; III) patients may contribute by donating their DNA sample to the bank thus increasing the number of collected DNA samples for future studies; IV) the public registry would be available to contribute knowledge which can be generalised regardless results from genetic studies. (Table 3)

## Conclusion

IgAN is a complex disorder with both genes and environment contributing in the pathogenesis. A Biobank, depository of a large number of DNA samples from IgAN patients and their relatives, together with a database including their personal and clinical data, has a crucial role in the genetic dissection of IgAN. Such an organization favours and stimulates collaborative studies. The constitution of a well organized and multi-centre biobank, an adequate choice with respect to design strategies and improved genotyping methods could greatly enhance the current understanding of the molecular genetic basis of this disease. Finding complex disease genes may allow us to determine which subjects are at risk of IgAN before that genetic susceptibility is converted into disease. In addition, the identification of such genes should reveal more about the molecular pathway causing the disease, thus suggesting new and relevant targets for more efficient drug treatment.

## Availability and requirements

A password is not required to search the IgAN Consortium website database, the password is only necessary to insert and to modify data [17].

## Competing interests

The author(s) declare that they have no competing interests.

## Authors' contributions

FPS founded the European IgAN Consortium. FS, AA, JF, EA, GMG and GMF contributed in organizing the IgAN Consortium Biobank. GC, DDT, MF, DP, PRM and DK collected DNA samples, clinical and personal data of the enrolled subjects. Moreover they provided the management of the database. KZ, LZ and LB contributed in the molecular genetic study design. The material submitted has been approved by all authors, has not been previously reported, and is not under consideration for publication elsewhere.

## Pre-publication history

The pre-publication history for this paper can be accessed here:



## Supplementary Material

Additional File 1 Appendix 1: The scientific committee represented by the principal investigators of the IgAN Consortium research units Click here for file

## Figures and Tables

**Table 1:** Collection of DNA samples from IgAN patients, relatives and apparently healthy controls obtained by the IgAN Consortium.

**Partner No.**	**Local site**	**IgAN families No.**	**Trios No.**	**Biopsy-proven IgAN patients No.**	**Controls No.**
1	Bari, Italy	32 (43*)	91	456	215
2	Brescia, Italy	31(5*)	47	360	204
3	Trieste, Italy	4(3*)	6	116	221
4	Aachen, Germany	2(8*)	9	62	200
5	Thessaloniki, Greece	3(4*)	6	74	200

**Total samples**		72(63*)	**159**	**1068**	**1040**

*Number in brackets represents additional suspected IgAN families. These are families in which one member is affected by biopsy-proven IgAN and others -not biopsied have recurrent macroscopic hematuria episodes in concomitance of upper respiratory tract infections or persistent microscopic hematuria.

**Table 2:** Distribution of pairs of relatives in 72 IgAN families according to genetic relationship and IgAN.

	**IgA Nephropathy status**				
	
**Relationship**	**A/A**	**A/P**	**A/N**	**N/N**	**Total pair**
**Spousal**	0	2	25	35	62
**Parent-offspring**	20	19	147	211	397
**Sibling**	44	32	168	185	429
**Half-sibling**	0	0	4	0	4
**Granparental**	0	4	5	89	98
**Avuncular**	8	22	170	382	582
**Cousin**	5	6	40	238	289
**Other**	17	9	141	545	712

**Total Pairs**	**94**	**94**	**700**	**1685**	**2573**

(A) affected subjects with biopsy-proven disease; (P) subjects probably affected by IgAN when persistent microscopic hematuria without biopsy-proven glomerulonephritis, or chronic renal insufficiency or ESRD treated with hemo or peritoneal dialysis, or renal transplantation after biopsy-proven IgAN or unknown glomerulonephritis, or death for chronic uremia, occurred; (N) non-affected subjects in the absence of urinary abnormalities.

**Table 3:** Information in the Data DNA Bank

• Descriptive information
Brief title
Brief summary
Condition or disease
Availability for single-patient or expanded-access use
• Recruitment information
Overall studies status
• Location and Contact information
• News
