# Supplementary material for: The IgA nephropathy Biobank. An important starting point for the genetic dissection of a complex trait
Source: BMC Nephrol. 2005 Dec 5;6:14. doi: 10.1186/1471-2369-6-14 (PMC1318455; doi:10.1186/1471-2369-6-14)
Supplement: Additional File 1 — Appendix 1: The scientific committee represented by the principal investigators of the IgAN Consortium research units [file 1471-2369-6-14-S1.doc]

Appendix 1

| **Project coordinator:**   - **Name: Francesco Paolo Schena** - **Organization: University of Bari** - **Postal address: Piazza G. Cesare 11, 70124 Bari, Italy** - **Telephone: 0039 080 5478869** - **Telefax: 0039 080 5575710** - **e-mail:** [**fp.schena@nephro.uniba.it**](mailto:fp.schena@nephro.uniba.it) |
| --- |
| **Principal investigators of the participating Research Units:**   - **Francesco Scolari- Università degli Studi di Brescia, Piazzale Spedali Civili 1,** **Brescia, Italy.** - **Antonio Amoroso – Dipartimento di Genetica, Biologia e Biochimica Università degli Studi di Torino, Via Santena 19, 10126 Torino, Italy.** - **Juergen Flöege – Division of Nephrology and Immunology, University of Aachen, Pauwelstrasse 30, 52057 Aachen, Germany.** - **Klaus Zerres- Institute of Human Genetics, University of Aachen, Pauwelstrasse 30, 52057 Aachen, Germany.** - **Efstathios Alexopoulos – Department of Nephrology, Aristotelian University of Thessaloniki, Papanastasiou 50, 54642 Thessaloniki, Greece.** - **Leopoldo Zelante – Genetic Unit, IRCCS-Casa Sollievo della Sofferenza, Viale Cappucini 1, 71013 S. Giovanni Rotondo, Italy.** |
